# Pro-Resolving Lipid Mediators (SPMs) and Their Actions in Regulating miRNA in Novel Resolution Circuits in Inflammation

**DOI:** 10.3389/fimmu.2012.00298

**Published:** 2012-10-22

**Authors:** Antonio Recchiuti, Charles N. Serhan

**Affiliations:** ^1^Center for Experimental Therapeutics and Reperfusion Injury, Department of Anesthesiology, Perioperative and Pain Medicine, Brigham and Women’s Hospital, Harvard Medical School, Harvard Institutes of MedicineBoston, MA, USA

**Keywords:** resolution, resolvin, protectin, n-3 PUFA, lipoxin

## Abstract

Unresolved inflammation is associated with several widely occurring diseases such as arthritis, periodontal diseases, cancer, and atherosclerosis. Endogenous mechanisms that curtail excessive inflammation and prompt its timely resolution are of considerable interest. In recent years, previously unrecognized chemical mediators derived from polyunsaturated fatty acids were identified that control the acute inflammatory response by activating local resolution programs. Among these are the so-called specialized pro-resolving lipid mediators (SPMs) that include lipoxins (LX), resolvins (Rv), protectins (PD), and maresins (MaR), because they are enzymatically biosynthesized during resolution of self-limited inflammation. They each possess distinct chemical structures and regulate cellular pathways by their ability to activate pro-resolving G-protein coupled receptors (GPCRs) in a stereospecific manner. For instance, RvD1 controls several miRNAs of interest in self-limited acute inflammation that counter-regulate the mediators and proteins that are involved in inflammation. Here, we overview some of the biosynthesis and mechanisms of SPM actions with focus on the recently reported miR involved in their pro-resolving responses that underscore their beneficial actions in the regulation of acute inflammation and its timely resolution. The elucidation of these mechanisms operating *in vivo* to keep acute inflammation within physiologic boundaries as well as stimulate resolution have opened resolution pharmacology and many new opportunities to target inflammation-related human pathologies via activating resolution mechanisms.

## Acute Inflammation: A Protective Host Response that Can Turn Harmful

**Figure 1:**
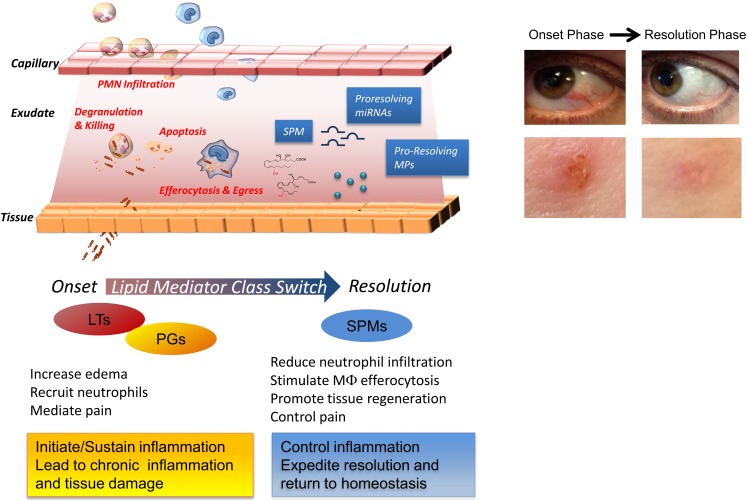
**Cellular and molecular mediators in acute inflammation and resolution**. Surgical intervention, tissue injuries, or microbial infections in vascularized tissues evoke a rapid acute inflammatory response characterized by a rapid exudate formation with edema, leukocyte infiltration, and serum proteins. Polymorphonuclear leukocytes (PMN) are among the first responders that fight microbes followed by monocytes that differentiate locally into pro-resolving macrophages (MΦs). Efferocytosis of apoptotic PMN and microbes by pro-resolving MΦs and subsequent egress via lymphatics are hallmarks of tissue resolution. Redness and swelling, two of the cardinal signs of inflammation, can be easily appreciated in the example of eye and skin inflammation shown in the right panel. A few days later, inflammation is almost completely resolved and homeostasis re-established. (Pictures were taken using a Lumix SZ7 digital camera). Lipid autacoids prostaglandins (PGs) and leukotrienes (LTs) are classical mediators of the onset phase of inflammation, promoting edema, PMN recruitment, and pain. By sustaining inflammation, PGs and LTs can lead to chronic inflammation and tissue damage. Specialized pro-resolving lipid mediators (SPMs) are biosynthesized within resolving exudates and proved to be very potent in reducing further PMN infiltration, stimulating non-phlogistic MΦ efferocytosis, promoting tissue regeneration, and controlling pain. Recent advances have demonstrated that specific miRNAs and microparticles can possess pro-resolving properties.

Acute inflammation is a defensive physiological response occurring in vascularized tissues to protect the host against injuries (Majno and Joris, 1996). This formidable ally manifests its important role, for instance, in the early phase after a microbial infection, when it fights against invading pathogens before the adaptive immune system is engaged (Abbas et al., 2011). The characteristic “cardinal signs” of inflammation, described by the Roman physician Celsus in the first century, *rubor* (redness), *tumor* (swelling), *calor* (heat), and *dolor* (pain), are the macroscopic manifestation of changes that occur at molecular and cellular levels in inflamed tissues. Tissue edema is one of the earliest events in the acute inflammatory response that arises from increased vascular permeability of the microvasculature (Figure 1). Leukocytes are then recruited at sites of inflammation and traverse postcapillary venules. Polymorphonuclear neutrophils (PMN) are among the first leukocyte responders that accumulate in the inflamed site. As they are the first line of defense of the innate immune system, these cells kill pathogens by engulfing them via phagocytosis and release of microbicidal proteins stored in their intracellular granules and reactive oxygen species into phagolysosomal vacuoles to kill invaders (Majno and Joris, 1996). Next, in experimental acute inflammation, mononuclear cells enter the inflammatory site. They can differentiate into macrophages (MΦs) and clear microbes, cellular debris, and apoptotic PMN by phagocytosis in a non-phlogistic process termed efferocytosis (Honn et al., 1989; Gordon, 2007; Serhan et al., 2007).

Ultimately, the clearance and efflux of phagocytes allow for resolution of the tissue and the return to homeostasis, namely catabasis (Figure 1). In order to maintain a healthy status, both the initiation of acute inflammation and its resolution must be efficient. Notably, it is not how often or how extensive an acute inflammatory reaction starts, but how effectively and quickly it resolves that determines whether the battle of inflammation is detrimental or the ideal favorable outcome for the host. Indeed, uncontrolled or unresolved inflammation is now recognized as a major driver of human pathologies, including arthritis, asthma, cancers, and cardiovascular diseases (Serhan, 2004; Serhan and Savill, 2005; Nathan and Ding, 2010). Given the high occurrence of these and many other diseases, understanding how acute inflammation resolves is of wide interest.

This review focuses on the specialized pro-resolving mediators (SPM) that are biosynthesized from essential polyunsaturated fatty acids (PUFAs), arachidonic acid (AA), eicosapentaenoic acid (EPA), and docosahexaenoic acid (DHA), namely lipoxins (LX), resolvins (Rv), protectins (PD), and maresins (MaR) and on their biosynthetic pathways, receptors, and miRNAs that act to control self-limited inflammation and promote its timely resolution. For readers interested in the biosynthesis of Rv and PD, this subject was recently reviewed in detail in Bannenberg and Serhan (2010), and the confirmation and total organic synthesis in Serhan and Petasis (2011).

## Resolution is an Active Process Controlled by SPM: Self-Limited Experimental System

At the histological level, resolution was well described by pathologists for more than 100 years as the time when the neutrophils that infiltrated the inflamed tissue sites leave or are lost from the site (Majno and Joris, 1996). Traditionally, resolution was thought to be a passive process, simply due to the attenuation/dissipation of chemotactic and pro-inflammatory signals. Our results (Serhan et al., 2000a; Levy et al., 2001), followed by those from many others worldwide (reviewed in a consensus report in Serhan et al., 2007) demonstrated that resolution is instead an *active process* orchestrated by special novel chemical mediators that *turn on* biochemical and cellular pathways to enable the return to homeostasis.

Lipid mediators (LM) from PUFA play essential roles in distinct phases of acute inflammation, with prostaglandins (PGs; Flower, 2006; Samuelsson, 2012) and cysteinyl leukotrienes (cysLTs) promoting early increase in vascular permeability and leukotriene (LT) B_4_ acting as a potent leukocyte chemoattractant (Samuelsson, 1983). Prostaglandins also contribute to fever and pain (von Euler, 1973). Chronic inflammation is widely viewed as an excess of pro-inflammatory mediators (Figure 1; Nathan and Ding, 2010). Results from our laboratory first demonstrated that the resolution phase is characterized by the active biosynthesis of specific LM that operate as *“resolution agonists”* to a) keep inflammation within physiological boundaries and b) expedite the complete return to homeostasis (Figure 1). The identification of this new array of LM was achieved using self-limited or naturally resolving acute inflammation models *in vivo* and a systems approach (Serhan et al., 2000a, 2002). The pharmacologic impact of the Rv and PD was reviewed in (Serhan and Chiang, 2008). This new array of LM is now recognized as a genus of SPM (Serhan and Chiang, 2008) that have two broad functions and are anti-inflammatory and pro-resolving via stimulating multi-level actions. Accruing evidence indicates that failure or disruption of the endogenous pro-resolution pathways governed by SPM can be detrimental and underlie some of the mechanisms of chronic inflammatory diseases (Gilroy et al., 1999; Karp et al., 2004; Schwab et al., 2007; Chan and Moore, 2010). SPM exert their potent dual anti-inflammatory and pro-resolving activities in the low nano- to microgram dose range when added back into experimental inflammatory disease models (Serhan and Chiang, 2008) and provide biotemplates for the design of novel therapeutics currently in clinical trials (see http://Clinicaltrials.Gov Identifier: NCT00799552). Therefore, harnessing these SPM may provide fascinating opportunities in the new and uncharted terrain of resolution pharmacology, with a substantial shift from a depletion pharmacology (i.e., via inhibitors, blockers, antagonists) toward a new approach based on resolution agonists that activate endogenous protective and clearance mechanisms.

Additional chemical mediators are operative in inflamed tissues to switch off leukocyte infiltration and restore their physiological functions. Among these are several cytokines (e.g., TGFβ, IL-10) that accumulate in resolving exudates (Bannenberg et al., 2005); glucocorticoids and the glucocorticoid-induced annexin-1 protein, which tune the inflammatory response and bring about homeostasis (for a recent review see (Perretti and Dalli, 2009)); and the transcription factor NF-κB, which also carries some anti-inflammatory properties (Lawrence et al., 2001). Moreover, inducing PMN apoptosis as well as lymphoid cells while stimulating their prompt removal by MΦs also can promote resolution (Honn et al., 1989; Ariel et al., 2006). Recent results indicate that small inhibitors of cyclin-dependent kinases fulfill this goal (Leitch et al., 2012) as do annexin-1 peptides (Perretti and Dalli, 2009). Therefore, the resolution process can be pharmacologically targeted.

Importantly, resolution is not synonymous with endogenous anti-inflammation. This is because, in order to be considered a “pro-resolver,” a chemical and/or molecular entity, in addition to serving as a “stop signal” for neutrophil trafficking and other cardinal signs of inflammation (e.g., swelling, pain), must also stimulate efferocytosis by MΦ, favor the antibacterial activities, and promote tissue repair and regeneration to achieve homeostasis. Along these lines, PGE_2_ can have anti-inflammatory properties in certain settings via stimulation of cAMP, but is not acting as pro-resolver since it does not enhance the uptake and clearance of apoptotic cells by MΦs (Kunkel et al., 1981). Also, although cyclooxygenase (COX) inhibitors as well as certain lipoxygenase (LO) inhibitors reduce some of the cellular events of the inflammatory reaction (e.g., edema formation, PMN recruitment, and pain), they dramatically impact the endogenous pro-resolution circuits and may delay or even derange this ideal outcome of acute inflammation and thus are “resolution toxic” (Gilroy et al., 1999; Schwab et al., 2007). In contrast, aspirin and glucocorticoids work synergistically with endogenous pro-resolution pathways (Perretti et al., 2002).

Complete resolution also requires the clearance of the remnants of damaged tissues and activated or apopotic cells, so-called microparticles (MPs). Originally viewed merely as empty vesicles, MPs are now recognized as “specialized shuttles” used by the organism to transfer bioactive molecules from cell to cell. Their role in inflammation and resolution is now being appreciated. Recently, the anti-inflammatory properties of a PMN-derived sub-population of MPs were uncovered, where they appear to signal to activate resolution mechanisms (Gasser and Schifferli, 2004; Dalli et al., 2008; Norling et al., 2011). Mimicking this new endogenous mechanism in resolution, novel human PMN-derived nanoparticles containing AT-RvD1 or a LXA_4_ stable analog, termed humanized pro-resolving nanomedicines, were prepared. These SPM-enriched nanohumanized particles possessed beneficial bioactivities in reducing acute inflammation *in vivo*, expediting resolution, and promoting wound healing (Norling et al., 2011). Hence, NPs can serve as mimetics of endogenous pro-resolution pathways (Figure 1).

Active resolution includes gene expression regulation of several soluble chemical mediators (e.g., cytokines, chemokines), receptors (e.g., Toll-like receptors), as well as transcription factors. An emerging line of investigation indicates that many genes are under tight control of miRNAs, short non-coding RNA molecules that act as translational repressors of mRNA transcripts (Bartel, 2009). They are involved in many physiological and pathological processes, including cell development, cancer (Iorio and Croce, 2009), and inflammation (Sheedy and O’Neill, 2008; Alam and O’Neill, 2011; O’Neill et al., 2011). Our recent results uncovered roles of miRNAs in self-limited inflammation and in the resolution phase, specifically, RvD1-G-protein coupled receptor-dependent gene networks in resolution of acute inflammation as part of the endogenous circuitry that controls this active process (Recchiuti et al., 2011; Krishnamoorthy et al., 2012).

## Identifying SPM in Resolution

An unbiased systems approach was taken to identify the endogenous SPM and decode their mechanisms of action in resolution. For this, the murine dorsal air pouch and self-limited acute inflammation was ideal because it permitted isolation of contained inflammatory exudates (Serhan et al., 2000a, 2002) and also enabled direct LM lipidomics of bioactive products, as well as their inactive precursors and further metabolites, proteomics, and analyses of cellular composition of the resolving exudate; namely the natural means by which inflammation returns to resolution and homeostasis. With this systems approach it was also possible to establish the local and temporal dissociation of LM biosynthesis (Bannenberg et al., 2005). For example, upon initiation of inflammation with TNF-α, there was a typical acute-phase response characterized by rapid PMN infiltration preceded by local generation of both PGs and LTs. Unexpectedly, the eicosanoids undergo what was termed earlier a “class switch” and the profiles of LM made within this milieu switched with time (Levy et al., 2001). Indeed, the potent chemoattractants LT were deactivated and 15-LO required for LX and Rv production was transcriptionally activated (Levy et al., 2001). Notably, the omega-3 essential fatty acids DHA and EPA are precursors of Rv, PD, and MaR, are rapidly carried into the exudates via plasma edema and are then made available for conversion for the congregated exudate cells (Kasuga et al., 2008). Of interest, this *LM class switching* is driven in part by COX-derived PGE_2_ and D_2_, via transcriptional regulation of enzymes involved in LX biosynthesis (Levy et al., 2001). Hence, the concept that “alpha signals omega,” namely the beginning signals the end in inflammation, was introduced by Sir John Savill and one of us to emphasize this finding (Serhan and Savill, 2005) to note that at time zero mediators are biosynthesized that signal to limit PMN influx and terminate the contained acute inflammatory response.

### What is a lipid mediator?

**Figure 2:**
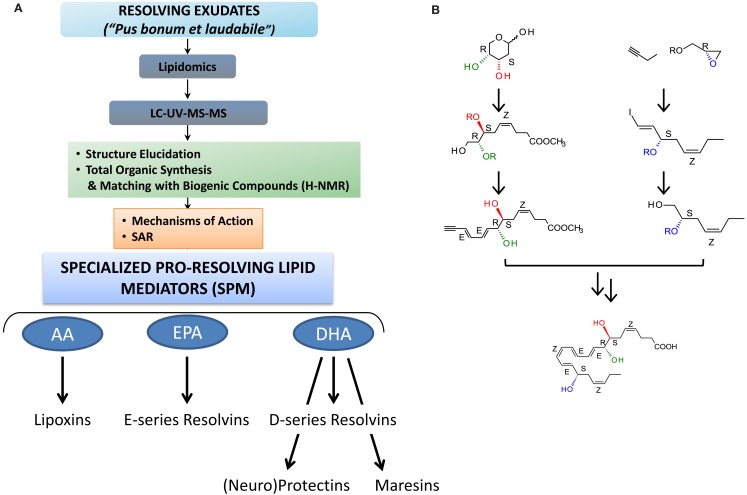
**Strategy for functional profiling of SPM in resolving exudates**. **(A)** During self-limited inflammation, murine exudates (a “good and laudable” pus according to ancient physicians; Majno, 1991) as well as human leukocytes biosynthesize SPM, which include the lipoxins, E-series resolvins, D-series resolvins, protectins (neuroprotectin D1), and maresins, which work to keep the inflammatory response within physiological boundaries and help to expedite the return to homeostasis. Functional profiling takes advantage of liquid chromatography-ultraviolet spectrometry-tandem mass spectrometry (LC-UV-MS/MS) for identifying and quantifying SPM. Gas chromatography-mass spectrometry (GC-MS) is also useful to provide additional information together with LC-UV-MS/MS to support structural identification and proposed structures. Retrograde analysis with biogenic synthesis using isolated human cells and total organic synthesis allows the assignment of chirality and double bond geometries using H-NMR with synthetic materials and matching studies (see text; Fiore et al., 1991; Serhan et al., 2000a, 2002, 2006, 2012; Sun et al., 2007; Spite et al., 2009a; Krishnamoorthy et al., 2010 for details). Bioactions of SPM are assessed in both animal models and human cell systems. They must be stereoselective and evident at concentrations/doses that are commensurate with the amount of SPM produced. **(B)** Example of RvD1 stereoselective total organic synthesis (reported in Sun et al., 2007); for further details (see Serhan and Petasis, 2011) for a recent review of organic synthesis.

To qualify as a LM, a product must be stereoselective in its actions and be produced in amounts that are commensurate with its potency and range of action (Serhan et al., 1996). Along this line, LX, Rv, and PD are present in human serum in pM to nM amount (e.g., LXA_4_, ∼1.4 nM; RvD1, ∼50 pM; RvE1, ∼0.5 nM; values from the Serum Metabolome Project; Psychogios et al., 2011; see also Oh et al., 2011 for RvE1 and Oh et al., 2012 for RvE2 values) in human peripheral blood samples from healthy donors. LM lipidomics using liquid chromatography-tandem mass spectrometry (LC-MS/MS) coupled with informatics permit profiling of closely related compounds and identification of new molecules. Retrograde synthesis, both biogenic and total organic, allows the complete elucidation of chemical structure, stereochemistry, and physical properties, along with the recapitulation of the *in vivo* biosynthetic pathway (for examples see Sun et al., 2007; Serhan et al., 2009). The matching/identification of LM is usually carried out with at least two different instruments and/or mobile phase solvent systems and the criteria to identify a known LM are the following: (a) LC retention time should match by coelution with the LM authentic standard; (b) UV chromophore should match the synthetic and authentic LM (i.e., λ_max_ and band shape); as well as (c) ≥6 diagnostic ions of tandem MS/MS spectrum (Figure 2). Also, the LC-MS/MS fragmentation mechanisms for the Rv and PD D1 and related DHA-derived products have been studied using deuterium-labeled compounds that facilitated their identification *in vivo* (Hong et al., 2007).

### Lipoxins

**Figure 3:**
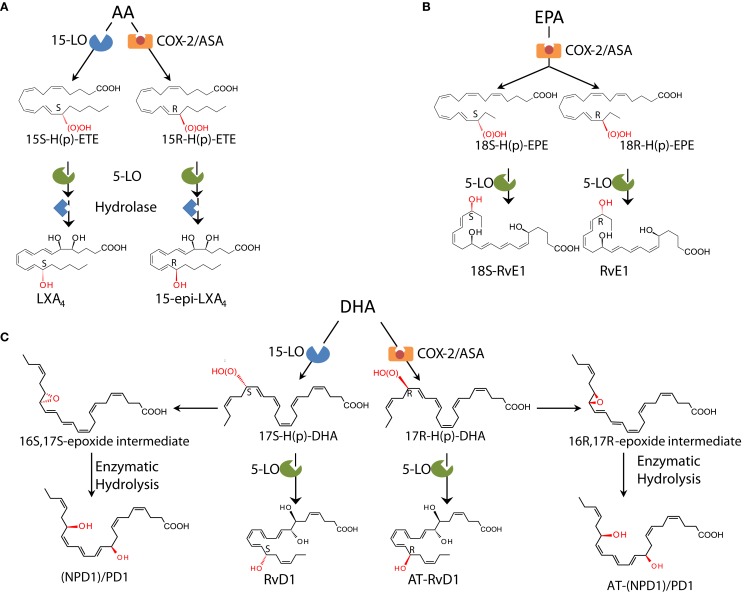
**Biosynthetic schemes of SPM**. **(A)** In humans, AA can be converted into 15S-H(p)-ETE through 15-LO and into 15R-H(p)-ETE by aspirin (ASA)-acetylated COX-2. Both intermediates can be further metabolized through 5-LO and enzymatic hydrolysis yielding LXA4 or 15-epi-LXA4. **(B)** E-series resolvins are biosynthesized via conversion of EPA by ASA-acetylated COX-2. Products of these reactions, 18S-H(p)-EPE and 18R-H(p)-EPE, are rapidly taken up by 5-LO and converted to 18S-RvE1 and RvE1. **(C)** The DHA metabolome includes several SPM biosynthesized via 15/5-LO and ASA-acetylated COX-2. Each SPM is biosynthesized via distinct biochemical routes involving stereocontrolled oxygenation, epoxide formation, and enzymatic hydrolysis. The main structures of key SPM and their biosynthetic routes (with precursors and main enzymes involved) are depicted (see text and Serhan and Petasis, 2011 for further details). The complete stereochemistry of each of these SPM is established, total organic synthesis achieved, and bioactions confirmed.

Lipoxin A_4_ and B_4_ were the first anti-inflammatory LM recognized to possess pro-resolving actions (Serhan et al., 1984a,b; Maddox et al., 1997; Takano et al., 1998; Godson et al., 2000). Although LXs were first isolated and identified in the 1980s in the Samuelsson laboratory (Serhan et al., 1984a), their potent bioactions were uncovered some years later with the identification of the aspirin-triggered LX (ATL; Claria and Serhan, 1995) and the design of ATL stable analogs (Serhan et al., 1995; *vide infra*), when it became clear that they act as “braking signals” of further PMN infiltration (Takano et al., 1998) and as potent stimuli for the non-phlogistic recruitment of monocytes (Maddox et al., 1997) and MΦ efferocytosis (Godson et al., 2000; recently reviewed in Serhan, 2005; Spite and Serhan, 2011). LXs are lipoxygenase interaction products derived from the enzymatic conversion of AA via transcellular biosynthesis during cell–cell interactions occurring during inflammation (Samuelsson et al., 1987). In humans, sequential oxygenation of AA by 15-LO and 5-LO, followed by enzymatic hydrolysis, leads to the biosynthesis of LXA_4_ and B_4_ in mucosal tissues, such as airways, gastrointestinal tract, and oral cavity (Edenius et al., 1990; Levy et al., 1993; Gronert et al., 1998; Figure 3 and reviewed in Romano (2010). Blood vessels represent a second site for LX biosynthesis, with the conversion of 5-LO-derived LTA_4_ into LXA_4_ and B_4_ by 12-LO in platelets (Serhan and Sheppard, 1990; Romano and Serhan, 1992; Romano, 2010).

### ATL: The first aspirin-triggered mediators

A third LX synthetic pathway is initiated by aspirin, the well-known derivative of salicylates, by acetylation of COX-2. This covalent modification shifts the enzyme activity from endoperoxidase to lipoxygenase-like, and COX-2 converts AA into 15R-HETE, which is a substrate of leukocyte 5-LO for the biosynthesis of 15R-epi-LXA_4_ and B_4_ (Claria and Serhan, 1995). Hence, among the non-steroidal anti-inflammatory drugs (NSAID), aspirin has the unique capability to “jump start” resolution by its ability to trigger endogenous biosynthesis of so-called “aspirin-triggered” LX (Figure 3) Notably, ATL produced *in vivo* in human subjects taking aspirin (Chiang et al., 2004) proved to mediate the local anti-inflammatory actions of low-dose aspirin in healthy individuals (Morris et al., 2009).

### SPM biosynthesized from omega-3 polyunsaturated fatty acids: Local mediators

The essential roles of omega-3 PUFA in health were evident in 1929 (Burr and Burr, 1929), and ω-3, also known as n-3 PUFA EPA and DHA, have beneficial effects in human diseases including potential antithrombotic, immunoregulatory, and anti-inflammatory properties (Iigo et al., 1997; De Caterina, 2011). Also, the Gruppo Italiano per lo Studio della Sopravvivenza nell’Infarto Miocardico-Prevenzione trial evaluated the effects of ω-3 PUFA supplementation with >11,000 patients surviving myocardial infarction taking >1 g of ω-3 PUFA daily along with recommended preventive treatments including aspirin, and reported a significant benefit with a decrease in cardiovascular death (GISSI-Prevenzione Investigators, 1999). It is believed that the actions of the major lipid of fish oil EPA (C20:5) are based upon (a) preventing conversion of AA to proinflammatory and prothrombotic eicosanoids; (b) serving as an alternate substrate for the five-series LTs that are less potent than four-series LTs; and (c) conversion by COX to three-series prostanoids (i.e., PGI_3_) that also maintain antithrombotic actions. These and other explanations offered (Iigo et al., 1997; Calder, 2009; De Caterina, 2011) have not been generally accepted because of the lack of molecular evidence *in vivo* and the high concentrations of ω-3 PUFA required to achieve putative “beneficial actions” in *in vitro* cell culture experiments.

To address the molecular basis for anti-inflammatory properties of ω-3 fatty acids, an unbiased LC-MS/MS-based informatics approach was developed to identify novel mediators generated from ω-3 precursors during acute inflammation *in vivo*. Using this approach, EPA and DHA were found to be enzymatically converted into novel potent LMs coined Rv, an acronym of *resolution phase interaction products*, because they: (a) are produced during cell–cell interactions occurring in the resolution phase of acute inflammatory response; (b) “stop” further neutrophil entry to sites of inflammation, and (c) reduce exudates (Serhan et al., 2000a, 2002, 2006; Hong et al., 2003; Bannenberg et al., 2005). Rv represented an entirely new family of mediators produced from the ω-3 fatty acids and importantly they appeared during the resolution phase via active biosynthetic processes. The biosynthesis of Rv gives rise to stereospecific local mediators that have potent actions and activate specific receptors.

### E-series resolvins

EPA-derived E-series Rv are endogenously biosynthesized *in vivo* in resolving murine exudates and in isolated human cells systems by isolated cells (e.g., endothelial cell-leukocyte interaction) and in whole blood (*vide infra*). The complete stereochemistry of the first member of this family, RvE1, has been established as 5S,12R,18R-trihydoxy-6Z,8E,10E,14Z,16E-EPA (Arita et al., 2005a). For further details on the total organic synthesis (see Serhan and Petasis, 2011). Within vascular endothelial cells, aspirin-acetylated COX-2 converts EPA into 18R-hydro(peroxy)-eicosapentaenoic acid (HEPE), which is rapidly taken up by activated leukocytes (e.g., PMN) and further metabolized into RvE1. Notably, quantitative chiral HPLC analysis indicated that the 18*R*-HEPE isomer was dominant to its epimer 18*S*-HEPE in human plasma from healthy subjects taking EPA (Oh et al., 2011). In contrast, human subjects who were administered aspirin before EPA had more 18*S*- than 18R-HEPE, indicating that aspirin might promote 18*S*-HEPE production as well as 18*R*-HEPE from ingested EPA (Oh et al., 2011). This 18*S*-HEPE can also be converted to epimeric RvE1 and RvE2 by human recombinant 5-LO and LTA_4_ hydrolase (LTA_4_H), known as pro-inflammatory LTB_4_-synthesizing enzymes (Oh et al., 2011). RvE1 is also produced *in vivo* through an aspirin-independent pathway via cytochrome P450-driven oxygenation of EPA (Serhan et al., 2000b). Of interest, RvE1 was also found to be produced by *Candida albicans* and appears to be involved in clearance of this organism (Haas-Stapleton et al., 2007). Thus RvE1 has multiple biosynthetic routes. RvE2 (5*S*,18-dihydroxy-EPE) is biosynthesized in resolving exudates and in human whole blood via reduction of 5*S*-hydroperoxy,18-hydroxy-EPE, an intermediate in the biosynthetic pathway of RvE1 (Tjonahen et al., 2006; Ogawa et al., 2009; Oh et al., 2012; Figure 3).

### D-series resolvins

Earlier investigations using LC-MS/MS lipidomics of resolving exudates from mice given DHA and aspirin provided the first evidence of novel endogenous routes that lead to the formation of 17-hydroxy-containing mediators. Gaining information on how human tissue and cells may produce D-series Rv involved the *in vitro* recapitulation of biosynthetic pathways using isolated human cells and recombinant enzymes establishing potential origins of novel compounds isolated from resolving exudates *in vivo*. Along these lines, hypoxic human endothelial cell COX-2 converted DHA to 13-hydroxy-DHA, which switched with ASA to 17R-HDHA. Human neutrophils transformed 17R-hydroxy-DHA into two series of di- and trihydroxy products; one initiated via oxygenation at carbon 7 and the other at carbon 4 (Serhan et al., 2002). The conversion of 17*R*-HDHA by human PMNs displayed similar features as those established for the conversion of AA to LTB_4_ or LXs as well as the 18*R* series of EPA products. These were termed the “aspirin-triggered” D-series Rv (Serhan et al., 2002). Remarkably, in the absence of aspirin, D-series Rv carrying the 17S-hydroxy group were identified in murine exudates and isolated human cells (Serhan et al., 2002; Hong et al., 2003). The enzymatic pathway leading to the formation of 17*S*- and 17*R*-RvD1 is shown in Figure 3. Following the complete organic synthesis, the stereochemistry of 17S-, 17R-RvD1, and RvD2 were established as 7S,8R,17S-trihydroxy-4Z,9E,11E,13Z,15E,19Z-DHA (17S-RvD1), 7S,8R,17R-trihydroxy-4Z,9E,11E,13Z,15E,19Z-DHA (17R-RvD1; Sun et al., 2007), and 7*S*, 16*R*, 17*S*-trihydroxy-4*Z*, 8*E*, 10*Z*, 12*E*, 14*E*, 19*Z*-DHA (RvD2; Spite et al., 2009a). Additional members of this family were identified (RvD3–RvD6). Each of these arises by similar biosynthetic routes, but has distinct chemical structures and potentially additional bioactions that are now being unveiled (Chiang et al., 2012). Importantly, both RvE1 and RvD1 were identified in circulating blood of healthy donors by (Psychogios et al., 2011) as part of the Serum Metabolome Project.

### The (neuro)protectins

In addition to D-series Rvs, DHA also serves as precursor of a new family of LM characterized by a conjugated triene system and two alcohol groups called PD. The name PD accounts for their protective actions observed in neural tissues and within the immune system, while the prefix neuro PD gives the tissue localization and site of action. The structure of the founding member of this family, PD1, was first disclosed in a report on the isolation and elucidation of Rv (Serhan et al., 2002; Hong et al., 2003), and its complete stereochemistry later established as 10*R*,17*S*-dihydroxy-docosa-4*Z*,7*Z*,11*E*,13*E*,15*Z*,19*Z*-hexaenoic acid (Serhan et al., 2006). In addition to PD1, several stereo- and positional isomers that also possess lower bioactivity than PD1 were identified in human and mouse tissues. These include 10S,17S-diHDHA, 4*S*,17*S-*diHDHA, 7*S*,17*S*-diHDHA, and 22-hydrox-10,17*S*-docosatriene (a putative inactivation product of PD1; Serhan et al., 2002; Hong et al., 2003). The geometry of the double bonds in PD1, their positions during biosynthesis, and chirality of C10 indicate that PD1 biosynthesis proceeds through a C16(17)-epoxide intermediate and requires specific enzymatic steps to generate the potent bioactive molecule from DHA, as confirmed by the isolation of alcohol trapping products as well as two vicinal diol 16,17-docosatrienes as minor products of PD1 biosynthesis (Hong et al., 2003; Serhan et al., 2006). Recently, a novel aspirin-triggered COX-2 driven pathway was reported that biosynthesizes the 17R-epimeric form of PD1 from DHA (Marcheselli et al., 2003; Figure 3). The total organic synthesis and complete stereochemical assignment of AT-PD1 (10R,17R- dihydroxy-docosa-4*Z*,7*Z*,11*E*,13*E*,15*Z*,19*Z*-hexaenoic acid) were recently achieved. Both PD1 and AT-PD1 reduced leukocyte infiltration in murine peritonitis, reduced PMN transmigration with endothelial cells, and enhanced efferocytosis of apoptotic PMN by human MΦ (Serhan et al., 2006). These are the hallmark actions of a SPM carrying both anti-inflammatory and pro-resolving actions demonstrable both *in vitro* and *in vivo*.

### Maresins

MΦs have pivotal roles in orchestrating the return to homeostasis (Gordon, 2007) and biosynthesize SPM that enhance their pro-resolving and homeostatic functions. For example, MΦ ingesting apoptotic cells initiate the biosynthesis of LXA_4_, RvE1, and PD1 but not LTB_4_ (Freire-de-Lima et al., 2006; Schwab et al., 2007). In addition, a new family of SPM biosynthesized by MΦs was identified (Serhan et al., 2009). Unbiased LM LC-MS/MS-based metabololipidomics during self-limited peritonitis led to the identification of a novel pathway that converted DHA into 14-hydroxy DHA (HDHA). Experiments with 12/15-LO^−/−^ mice or with LO inhibitor confirmed that 14-HDHA production was via a DHA carbon 14 lipoxygenation pathway. Freshly prepared 14-H(p)DHA is rapidly converted by isolated human and mouse MΦ into a new set of bioactive products, whose molecular structure was established (Serhan et al., 2009) and recently confirmed by matching of biogenic material with those prepared by total organic synthesis (Serhan et al., 2012). The major product of this new pathway proved to be 7,14-dihydroxydocosa-4*Z*,8*E*,10E,12Z,16*Z*,19*Z*-hexaenoic acid, denoted MaR (*ma*crophage mediator in *res*olving *in*flammation) 1 (first in the family; MaR1; Serhan et al., 2009). Similar to that of other potent SPM, MaR1 biosynthesis proceeds via an epoxide intermediate, specifically the MaR1 13,14-epoxide intermediate, that was demonstrated by trapping experiment and with ^18^O-containing molecular oxygen O_2_ that opens during enzymatic conversion to MaR1 keeping the double bond geometry and chirality of carbon 7 and 14.

In addition to MaR1 in resolving murine exudates, a novel double dioxygenation product was isolated and identified, 7*S*,14*S*-dihydroxydocosa-4*Z*,8*E*,10*Z*,12*E*,16*Z*,19*Z-*hexaenoic acid (denoted 7*S*,14*S*-diHDHA), formed by consecutive lipoxygenation of 14-HDHA, was also identified using molecular oxygen incorporation, and proved bioactive but less potent in activity than MaR1 in stimulating efferocytosis with human cells (Serhan et al., 2009, 2012). MaR1 and RvE1 are also potent stimulators of organ regeneration using a planaria regeneration system (Serhan et al., 2012). Hence, SPM are primordial molecules that signal from the inflammatory site to generate the aftermath of inflammation and tissue injury.

## GPCRs for SPM in Anti-Inflammation and Resolution

### GPCRs for LXs

The first evidence for receptor-mediated actions of LXA_4_ arises from studies with Santosh Nigam when he was on sabbatical in the Serhan Lab at BWH in the late 1980s, which demonstrated stimulation of rapid lipid remodeling and pertussis toxin (PTX)-sensitive release of arachidonate in PMN treated with LXA_4_ (Nigam et al., 1990). To examine the molecular basis of these actions, synthetic [11,12-^3^H]-LXA_4_ was prepared and used to demonstrate specific and reversible binding to intact human PMN with a *K*_d_ ∼ 0.5 nM. [^3^H]-LXA_4_ binding was stereoselective as LXB_4_, LTB_4_, 6S-LXA_4_, or 11-trans-LXA_4_ did not compete for LXA_4_ binding, while cysteinyl LT C_4_ and D_4_ partially displaced bound labeled LXA_4_ (Fiore et al., 1992). Screening of cDNA clones from differentiated HL60 human cells lines led to the identification of formyl peptide receptor like-1, a homolog of formyl receptor, as putative LXA_4_ GPCR (Fiore et al., 1994). This receptor has recently been coined ALX/FPR2 by the international nomenclature committee in light of its high affinity for LXA_4_ (Ye et al., 2009).

Human FPR2/ALX is highly expressed in myeloid cells and at a lower extent in lymphocytes, dendritic cells, and resident cells (Chiang et al., 2006). Orthologs of the human FPR2/ALX have been identified in mice (Takano et al., 1997) and rats (Chiang et al., 2003). In addition to LXA_4_, FPR2/ALX is activated by the glucocorticoid-induced protein annexin-1 and its N-terminal peptides (Perretti et al., 2002), representing the prototype of GPCR able to coordinate anti-inflammatory and pro-resolving activities of both lipid and peptide ligands. Genetic manipulation of ALX/FPR2 and its ortholog in mice has provided evidence for the essential role of this GPCR in controlling immune responses. Indeed, myeloid-driven overexpression of human FPR2/ALX in transgenic mice resulted in a reduced neutrophil infiltration during zymosan-induced peritonitis (Devchand et al., 2003), whereas ALX/FPR2^−/−^ mice have an exacerbated inflammatory phenotype and delayed resolution (Dufton et al., 2010).

More strikingly, ATL and FPR2/ALX expression levels dictate both the magnitude and duration of acute inflammation in humans (Morris et al., 2010). Hence, mechanisms that regulate this expression are of wide interest. Recent results from Simiele et al. (2012) uncovered the molecular basis of ALX/FPR2 transcription machinery, with the identification of the core promoter sequence, the elucidation of transcription factors and epigenetic mechanisms that regulate promoter activity, and the identification of the first inheritable SNP that impairs promoter activity in individuals at high cardiovascular risk. Notably, LXA_4_ upregulates ALX/FPR2 levels by activating its promoter, suggesting an additional mechanism by which LXA_4_ exerts its bioactivities (Simiele et al., 2012). This is particularly relevant in considering LX roles in stimulating resolution. In addition, earlier studies demonstrated that radiolabeled 15-epi-LXA_4_ binds at cysteinyl LT receptor 1 (CysLT1) with equal affinity to LTD_4_, providing additional molecular mechanisms for ATL dampening CysLT signals in the vasculature as well as regulating leukocyte trafficking via ALX/FPR2 (Gronert et al., 2001).

### GPCRs for E-series resolvins

At least two GPCRs are involved in mediating RvE1 actions, namely ChemR23 and BLT1 (Arita et al., 2005a, 2007). RvE1 binding to ChemR23 was assessed with [^3^H]-labeled RvE1, which was prepared by catalytic hydrogenation from synthetic diacetylenic RvE1. [^3^H]-RvE1 bound to ChemR23 transfectants with high affinity (*K*_d_ = 11.3 ± 5.4 nM) and stereoselectivity, since RvE1 biogenic precursors EPA and 18*R*-HEPE did not compete with [^3^H]-RvE1. Also, the synthetic peptide fragment (YHSFFFPGQFAFS) derived from human chemerin that was earlier reported to be a ligand for this same receptor (Wittamer et al., 2003) displaced [^3^H]-RvE1 binding by ∼70% when tested at 10 μM concentration, suggesting that RvE1 and chemerin share recognition sites on ChemR23 (Arita et al., 2005a; Ohira et al., 2009). [^3^H]-RvE1 specific binding was also demonstrated with membrane fractions isolated from human PMN. Radiolabeled RvE1 bound human PMN with *K*_d_ of ∼50 nM and was displaced by homoligand RvE1 (*K*_i_ ∼ 34 nM), LTB_4_ (*K*_i_ = 0.08 nM), and LTB_4_ receptor 1 (BLT1) selective antagonist U-75302 (*K*_i_ = 1.5 nM), but not by the chemerin peptide (Arita et al., 2007). These results strikingly demonstrated that RvE1 binding sites are pharmacologically distinct from ChemR23 on human PMN and prompted us to investigate whether RvE1 binds to LTB_4_ receptors.

In these studies, Arita et al. found that [^3^H]-RvE1 also gave high affinity binding to recombinant BLT1 (*K*_d_ ∼ 45 nM) that was competed by unlabeled LTB_4_ (*K*_i_ = 3 nM). In contrast, BLT2-overexpressing cells did not show [^3^H]-RvE1 binding at concentrations up to 10 nM. These results clearly demonstrated that RvE1 binds to BLT1 on human PMN and acts as a partial agonist to attenuate LTB_4_ incoming signals in both mouse and human leukocytes (Arita et al., 2007).

Profiling for tissue distribution of human ChemR23 showed expression of this GPCR in brain, kidney, cardiovascular, gastrointestinal, and myeloid tissues (Arita et al., 2005a). More recently, direct evidence for ligand-receptor interactions of RvE1 and its epimer 18S-RvE1 was provided using ChemR23 and BLT1 β-arrestin cells. In this system, cells were engineered to co-express a β-arrestin protein tagged with an inactive moiety of β-galactosidase enzyme together with a candidate GPCR fused to the short Pro-Link peptide derived from β-galactosidase. In the presence of ligand, in this context RvE1 activates GPCR interacts with β-arrestin, bringing to proximity two inactive portions of β-galactosidase and reconstituting the fully active enzyme. The activity of this enzyme, which is stoichiometrically dependent on GPCR-ligand interaction, is monitored with a chemiluminescence detection system. With ChemR23 β-arrestin cells, 18*S*-RvE1 dose-dependently activated ChemR23 receptor, with EC_50_ (∼6.3 pM) lower than that obtained with RvE1 (∼0.14 nM). 18*S-*RvE1 also antagonized LTB_4_-mediated BLT1 activation with higher potency and efficacy than RvE1 in BLT1 β-arrestin cells (Oh et al., 2011). Hence, RvE1 and 18*S*-RvE1 can share the same site(s) of specific binding to human ChemR23 as well as BLT1 and thus suggest location-dependent mechanisms in their signaling capabilities.

RvE2 exerts potent and cell-specific bioactions on human leukocytes (Tjonahen et al., 2006; Oh et al., 2012). Recently, tritium-labeled [^3^H]-RvE2 was synthesized and gave comparable *K*_d_ (∼25 nM) with other SPM in isolated human PMN. In addition, using ChemR23 and BLT1 β-arrestin cells, RvE2 was found to share, at least in part, receptors with RvE1 (Oh et al., 2012).

### GPCRs for D-series resolvins

**Figure 4:**
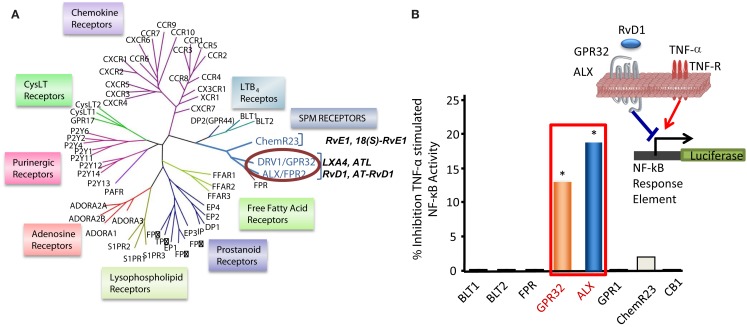
**Identification of RvD1 GPCR**. **(A)** Phylogenetic tree shows similarities in the amino acid sequences of human GPCRs closely related to LXA_4_, RvE1, and LTB_4_ receptors (left panel). Cluster was generated with the ClustalW2 software (www.ebi.ac.uk/Tools/clustalw2). Protein sequences were deduced from the NCBI database and receptor nomenclature followed the IUPAR classification for GPCR. **(B)** For functional screening and identification of RvD1 receptor, GPCRs were cloned in pcDNA3 vector and overexpressed in human HeLa cells cotransfected with pNF-κB luciferase plasmid. Cells were treated with RvD1 and TNF-α (see inset in the right panel). RvD1 reduces TNF-α-stimulated NF-κB activation in DRV1/GPR32 and ALX/FPR2 receptor-overexpressing cells, while cells transfected with other related GPCRs (e.g., BLT1, BLT2, CB1, GPR-1, FPR, and ChemR23) did not significantly inhibit TNF-α-induced NF-κB luciferase activity on addition of the ligand RvD1. The results illustrated are expressed in luminescence units subtracted from pNF-κB and pcDNA3 empty vector (*P < 0.05 vs. BLT2-transfected cells).

RvD1 activates its own GPCR and does not activate ChemR23. RvD1 exerts specific bioactivities on human PMN, namely reduction of F-actin polymerization, that are inhibited by PTX but not cholera toxin, whereas it did not stimulate Ca^2+^ release nor activate cAMP in human PMN (Krishnamoorthy et al., 2010). For the purpose of investigating direct binding of RvD1 to human PMN, [^3^H]-RvD1 was prepared by catalytic hydrogenation of synthetic [13, 14]-acetylenic RvD1 methyl ester by custom tritiation (American Radiolabel; Krishnamoorthy et al., 2010). This procedure was followed by de-esterification and isolation of the RvD1 label using RP-HPLC. [^3^H]-RvD1 specifically bound to human PMN with high affinity (*K*_d_ ∼ 0.17 nM) and was competed by homoligand cold RvD1 (100%) and LXA_4_ (∼60%) but not the ALX-ligand annexin-1-derived Ac2-12 peptide. In parallel, [^3^H]-RvD1 also showed specific binding with human monocytes (Krishnamoorthy et al., 2010). Since RvD1 counteracts TNF-α *in vivo*, luciferase-based reporter system (Arita et al., 2005a) was employed in functional screenings to assess the ability of selected GPCRs (Figure 4) to transduce RvD1 signals that block NF-κB activity in response to TNF-α.

**Figure 5:**
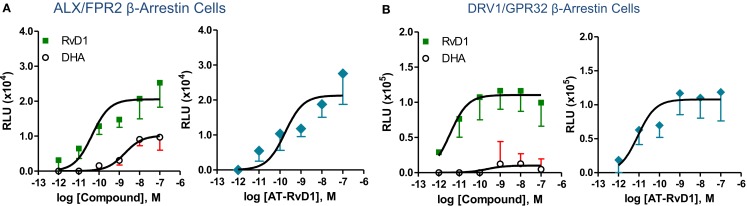
**Structure-activity relationship of RvD1, AT-RvD1, and DHA on ALX/FPR2 and DRV1/GPR32 receptors**. Activation of ALX/FPR2 and DRV1/GPR32 was determined using the β-arrestin cell system. This system is engineered by stably expressing the target ALX/FPR2 or DRV1/GPR32 tagged with the β-galactosidase Pro-Link peptide. Cells also co-expressed the β-arrestin protein linked to the β-galactosidase EA fragment. In the presence of ligand, activated GPCR interacts with β-arrestin, bringing to proximity the EA and Pro-Link fragments, forming a functional enzyme. β-galactosidase activity is measured by adding the substrate and generating a chemiluminescent signal that is stoichiometrically associated to ligand dependent GPCR activation (see text and Krishnamoorthy et al., 2010 for further details). Dose-response curves show activation of ALX/FPR2 **(A)** and DRV1/GPR32 **(B)** receptors by RvD1, AT-RvD1, but not DHA determined using the β-arrestin cell system. Results are mean (±SEM) from *n* = 4 to 6. RLU, relative luminescence unit.

Phylogenetically related GPCR linked to inflammation and chemoattraction were overexpressed into HeLa cells together with a reporter vector consisting of NF-κB promoter sequence linked to the luciferase gene. RvD1 significantly reduced TNF-α-stimulated NF-κB response in cells overexpressing either the LX receptor ALX/FPR2 or the orphan, GPR32, but not other GPCRs (e.g., BLT1, BLT2, CB1, GPR-1, FPR, and ChemR23; Krishnamoorthy et al., 2010). Moreover, RvD1 dose-dependently activated ALX/FPR2 and GPR32 in recombinant β-arrestin cells with EC_50_ in the low picomolar range (EC_50_ ∼ 1.2 pM for ALX/FPR2; 8.8 pM for GPR32; Figure 5). In contrast, RvD1 did not activate RvE1 receptor ChemR23, demonstrating the high selectivity of these ligands for their specific GPCR (Krishnamoorthy et al., 2010). In comparison, at equimolar concentrations, RvD1, its epimer AT-RvD1, RvD1-carboxy-methyl ester, and a metabolically more stable analog 17 (R/S)-methyl RvD1-ME activated both ALX/FPR2 and GPR32 with similar potencies and EC_50_, whereas the biosynthetic precursor native DHA was not active with GPR32 and ALX/FPR2 in this concentration (Krishnamoorthy et al., 2012). Of interest, the known anti-inflammatory ALX/FPR2 agonist compound 43 identified by traditional medicinal chemistry screening also activated GPR32 (EC_50_ ∼ 2.2 pM) and ALX/FPR2 (EC_50_ ∼ 2.0 pM) in β-arrestin cells but not the ADP receptor P2Y_12_ (Krishnamoorthy et al., 2010). Hence, RvD1, AT-RvD1, and the derivatives carboxy methyl ester and 17(R/S)-RvD1 directly activate ALX/FPR2 and GPR32, hereafter referred to as RvD1 receptor (DRV1) following the IUPAC recommendations for receptor nomenclature (Brink et al., 2003; Figure 5). Overexpression of either ALX/FPR2 or GPR32 in human MΦs gave further enhancement of efferocytosis in response to RvD1, while knockdown of ALX/FPR2 or DRV1/GPR32 determined a decrease in RvD1-stimulated phagocytosis response (Krishnamoorthy et al., 2010).

In keeping with this, Norling et al. (2012) demonstrated that the ability of RvD1 to reduce human PMN-endothelial cell interactions is absolutely dependent on ALX/FPR2 and DRV1/GPR32. Interestingly, actions of low concentration (1 nM) RvD1 were dampened by DRV1/GPR32 blocking antibody, whereas at high concentrations (10 nM) they appeared ALX/FPR2-specific. These two receptors also have distinct responses in an activated cell environment in that upon activation human PMN rapidly mobilized ALX/FPR2 stored in secretory granules, but not DRV1/GPR32, to the cell membrane. In addition, in ALX/FPR2 knockout mice RvD1 did not exert anti-inflammatory (e.g., stop PMN infiltration) nor pro-resolving (e.g., enhancing MΦ efferocytosis) actions (Norling et al., 2012). Hence, specific GPCRs selectively mediate RvD1 actions with ALX/FPR2 being rapidly upregulated in PMN that are exposed to pro-inflammatory stimuli and DRV1/GPR32 possibly conveying more homeostatic functions. With respect to cell and tissue distribution, ALX/FPR2 is present on leukocytes and resident cells (including MΦ, synovial fibroblasts, mesangial cells, endothelial, and epithelial cells; Krishnamoorthy et al., 2010). Human DRV1/GPR32 was identified in peripheral blood leukocytes and arterial and venous tissues using a cDNA array. It is mostly abundant on PMN, monocytes, and macrophages and is also present on vascular endothelial cells (Krishnamoorthy et al., 2010). The murine ortholog of DRV1/GPR32 is currently not known but is present in chimpanzees. Regulatory mechanisms of DRV1/GPR32 are of interest while those of ALX/FPR2 have recently been uncovered (Simiele et al., 2012). Although specific receptors for RvD2, RvD3, and RvD4 have not yet been identified, the stereoselective actions of RvD2 were inhibited by PTX (Spite et al., 2009a), implicating the involvement of GPCRs. More recently, Chiang et al. (2012) reported activation of RvD1-receptor DRV1/GPR32 by RvD5, which is related biosynthetically to RvD1, with the recombinant human DRV1.

### GPCRs for neuro (N)PD1/PD1

Biosynthesis of (N)PD1 occurs in neural tissues in response to injury, ischemia-reperfusion, and exposure to β-amyloid peptides (Marcheselli et al., 2003; Mukherjee et al., 2004; Bazan, 2007). In addition (N)PD1 shows protective anti-inflammatory and pro-resolving actions within the immune system (Serhan et al., 2002; Hong et al., 2003). Hence, it was of interest to determine the molecular basis of (N)PD1/PD1 actions. Specific binding of tritium-labeled (N)PD1 obtained by catalytic tritiation of synthetic 15-acetylenic NPD1 methyl ester was demonstrated with both retinal pigment cells (RPE) and human PMN. [^3^H]-(N)PD1 bound RPE with *K*_d_ ∼ 30 pmol/mg of cell protein. However, at high concentration of radio-ligand (>10 nM), non-specific binding was evident, in line with the highly hydrophobic nature of this compound. In these experiments, competitive binding studies with unlabeled ligand demonstrated 90–100% displacement for the free acid form of cold (N)PD1. In these studies (N)PD1-ME showed a lower affinity for binding sites and ∼74% displacement, while other structurally related omega-3 fatty acid-derived compounds (17*S*-hydroxy-DHA, RvE1, Δ15-*trans*-NPD1, and Δ15-*trans*-NPD1-carboxy methyl ester) gave only minimal or no displacement. Specific binding experiments with [^3^H]-NPD1 and isolated human PMN proved high affinity specific binding and showed a two-site fit for sites of high and low affinity binding (*K*_d_ ≈ 25 and 200 nM). Two other SPM that bind specific receptors on human PMN, namely LX A_4_ (LXA_4_) and RvE1, did not compete with [^3^H]-NPD1 binding on these cells (Marcheselli et al., 2010).

## How Do the Pro-Resolving Mediators Work?

By definition, SPM: (a) are generated within the resolution phase; (b) limit leukocyte infiltration; (c) enhance phagocytic activity of pro-resolving MΦ to remove apoptotic cells and/or microbes; (d) stimulate the clearance of PMN from mucosal surfaces and their anti-microbial actions. If a LM fulfills each of these bioactivities, then it belongs to the genus of SPM. At the cellular and molecular levels, SPM display distinct modes of action on PMN and monocyte/MΦs, which can be demonstrated with isolated cells. Each SPM (RvE1, RvE2, and RvD1) stimulates a rapid shape change of human leukocytes that reflects ligand-receptor responses and cytoskeletal events that ultimately limit the PMN to diapedesis *in vivo*, hence reducing inflammation and tissue damage (Dona et al., 2008; Kasuga et al., 2008; Oh et al., 2012) and prepare the macrophages to enhance phagocytosis of both apoptotic cells and microbes (Schwab et al., 2007; Ohira et al., 2009).

The best examples of these pro-resolving cellular mechanisms studied to date are for the Rv ligands RvE1 and RvD1. In the case of RvE1, its direct binding to the human recombinant GPCR denoted ChemR23, an RvE1 receptor, activates the receptor with the subsequent regulation of Akt intracellular signaling pathway and phosphorylation signaling of proteins that increase phagocytosis (Ohira et al., 2009). In the case of RvD1, the ligand reduces actin polymerization in isolated human PMN in a PTX-sensitive manner (Krishnamoorthy et al., 2010), diminishes surface expression of CD11b involved in leukocyte adhesion to endothelial cells (Krishnamoorthy et al., 2010) as does RvE1 (Dona et al., 2008), evokes a shape change and stops chemoattractant-initiated PMN migration observable at a single cell level with human cells isolated within microfluidic chamber systems (Kasuga et al., 2008). Of note, LXA_4_ also stops PMN chemotaxis *in vitro* (reviewed in Serhan, 2005), while it induces rapid activation of small GTPases and redistribution of cytoskeletal proteins (e.g., MYH9, F-actin) in human monocyte-derived MΦs during phagocytosis (Maderna et al., 2002; Reville et al., 2006). Hence, there appears to be a general mechanism on how pro-resolving molecules work to achieve their potent anti-inflammatory and pro-resolution actions, namely via SPM coupling to distinct high affinity receptors that evoke cell-type and specific intracellular pathways.

Besides these general actions, each SPM possesses additional specific activities (see Table 1; Fredman and Serhan, 2011). Given the important protective function of acute inflammation to fight infections or dangers from within and the need to safeguard the host from an uncontrolled reaction, it is not surprising that SPM have some bioactions overlapping in target tissues and specific cell types. In addition, the sites of biosynthesis for each SPM and the degree of cell distribution of their GPCRs may underlie selectivity and specificity of the pro-resolving system. In experimental models of inflammation and resolution, SPM proved to be stereoselectively active in the nano- to low microgram dose range to control inflammation, limit tissue damage, shorten resolution intervals, promote healing, and alleviate pain (Table 1). In order to define resolution in unbiased, quantitative terms, mathematical resolution indices were introduced for determining the cellular changes in exudates, i.e., *T*_max_, time point of maximum PMN infiltration (Ψ_max_); *T*_50_, time necessary to achieve 50% reduction in PMN number (Ψ_50_) from Ψ_max_; resolution interval (*R*_i_ = *T*_50_ − *T*_max_), time interval between *T*_max_ and *T*_50_ (Bannenberg et al., 2005). The introduction of resolution indices permits the evaluation of pro-resolution bioactions of endogenous chemical mediators or pharmacological agents (Schwab et al., 2007; Haworth et al., 2008; Navarro-Xavier et al., 2010). These results first demonstrate the possibility to pharmacologically manipulate resolution and stimulate resolution. Along these lines, results from the first human Phase I–II clinical trials demonstrated the safety and efficacy of a Rv analog that reduces both signs and symptoms of dry eye syndrome (http://Clinicaltrials.Gov Identifier: NCT00799552) and have moved forward to Phase III clinical trial with Celtic Therapeutics. Dye eye syndrome is a chronic illness commonly treated with the immune-suppressant cyclosporine, providing evidence that SPM have the potential to treat a broad array of human diseases.

**Table 1:** **Bioactions of SPM**.

SPM	Disease model	Mechanism of action	Reference
Lipoxin A4/ATL	Mouse/dermal inflammation	Inhibits neutrophil recruitment and vascular leakage	Takano et al. (1997)
	Mouse/dorsal air pouch	Inhibits neutrophil recruitment	Clish et al. (1999)
	Rabbit/periodontitis	Reduces PMN infiltration and prevents connective tissue and bone loss	Serhan et al. (2003)
	Mouse/peritonitis	Inhibits neutrophil recruitment and lymphatic removal of phagocytes	Bannenberg et al. (2005) and Schwab et al. (2007)
	Mouse/colitis	Attenuates pro-inflammatory gene expression and reduces severity of colitis, inhibits weight loss, inflammation, and immune dysfunction	Gewirtz et al. (2002)
	Mouse/asthma	Inhibits airway hyper-responsiveness and pulmonary inflammation	Levy et al. (2002)
	Mouse/cystic fibrosis	Decreases neutrophilic inflammation, pulmonary bacterial burden, and disease severity	Karp et al. (2004)
	Mouse/ischemia/reperfusion (I/R)	Attenuates hind limb I/R-induced lung injury. Detachment of adherent leukocytes in mesenteric I/R vessels. Reduces myocardial infarct size and area at risk in myocardial I/R	Scalia et al. (1997) and Chiang et al. (1999)
	Mouse/cornea	Accelerates cornea re-epithelialization, limits sequelae of thermal injury (i.e., neovascularization, opacity) and promotes host defense	Gronert et al. (2005)
	Mouse/angiogenesis	Reduces angiogenic phenotype: endothelial cell proliferation and migration	Fierro et al. (2002)
	Mouse/bone marrow transplant (BMT)	Protects against BMT-induced graft-versus-host diseases (GvHD)	Devchand et al. (2005)
	Rat/glomerulonephritis	Reduces leukocyte rolling and adherence; decreases neutrophil recruitment	Papayianni et al. (1995)
	Rat/hyperalgesia	Prolongs paw withdraw latency, reducing hyperalgesic index, and reduces paw edema	Svensson et al. (2007)
	Rat/pleuritis	Shortens the duration of pleural exudation	Bandeira-Melo et al. (2000)
	Mouse/tumor growth	Suppresses the growth of transplanted tumors in mice; inhibits angiogenesis	Chen et al. (2010)
	Mouse/allograft rejections	Prevents acute rejection of vascularized cardiac and renal allografts	Levy et al. (2011)
	Mouse/arthritis	Inhibits edema formation and PMN influx, reduces TNFα and LTB_4_ levels	Conte et al. (2010)
	Rat/acute pancreatitis	Reduces intercellular adhesion molecule 1 (ICAM-1) and NF-κB p65 expression in the pancreas, and expression of ICAM-1 in the lungs	Zhou et al. (2011)
	Zebrafish/mycobacterial infection	Reduces bacterial burden and growth; improves microbial containment by phagocytes	Tobin et al. (2012)
Resolvin E1	Mouse/dorsal air pouch	Inhibits neutrophil recruitment	Serhan et al. (2000a)
	Mouse/peritonitis	Inhibits neutrophil recruitment, regulates chemokine/cytokine production, and promotes lymphatic removal of phagocytes	Arita et al. (2005a), Bannenberg et al. (2005), and Schwab et al. (2007)
	Rabbit/periodontitis	Reduces PMN infiltration, prevents connective tissue and bone loss, promotes healing of diseased tissues, and promotes regeneration of lost soft tissue and bone	Hasturk et al. (2006, 2007)
	Mouse/retinopathy	Protects against neovascularization	Connor et al. (2007)
	Mouse/colitis	Decreases PMN recruitment and pro-inflammatory gene expression; improves survival and reduces weight loss; favors LPS-detoxification through induction of intestinal alkaline phosphatase	Arita et al. (2005b), Campbell et al. (2010), and Ishida et al. (2010)
	Mouse/asthma	Reduces IL-23 and IL-6, and increases IFNγ and LXA_4_ in lungs to dampen airway inflammation; decreases eosinophil and lymphocyte recruitment	Aoki et al. (2008, 2010) and Haworth et al. (2008)
	Mouse/obesity	Regulates adipokines and protects against liver steatosis	Gonzalez-Periz et al. (2009)
	Mouse/inflammatory pain	Inhibits spontaneous pain, and heat and mechanical hypersensitivity	Xu et al. (2010)
	Rat/cardiac ischemia/reperfusion injury	Reduces infarct size	Keyes et al. (2010)
	Mouse/allograft rejections	Prevents acute rejection of vascularized cardiac and renal allografts	Levy et al. (2011)
	Mouse/dry eye	Promotes tear production, corneal epithelial integrity, and decreases in inflammatory inducible COX-2. RvE1 inhibits keratocyte transformation to myofibroblasts and lowers the number of monocytes/macrophages	Li et al. (2010)
	Mouse/herpes simplex virus	Reduces severity of herpes simplex virus-induced ocular lesions, reduces angiogenesis, and stromal keratitis	Rajasagi et al. (2011)
Resolvin D1	Mouse/peritonitis	Inhibits neutrophil recruitment; shortens resolution interval; regulates miRNAs and target genes in resolving exudates; reduces LTB_4_, PGD_2_, PGF2_α_, and TXA_2_ in peritoneal exudates	Hong et al. (2003), Sun et al. (2007), Spite et al. (2009b), Recchiuti et al. (2011), Krishnamoorthy et al. (2012), and Norling et al. (2012)
	Mouse/*E. coli* (peritoneal) and *S. aureus* (skin) infection	Reduces bacterial titers and hypothermia; increased survival; enhances microbial containment and killing by phagocytes; lowers antibiotic requirement; shortens resolution interval	Chiang et al. (2012)
	Mouse/dorsal air pouch	Inhibits neutrophil recruitment	Serhan et al. (2002) and Hong et al. (2003)
	Mouse/kidney ischemia-reperfusion	Protects from ischemia/reperfusion-induced kidney damage and loss of function; regulates macrophages	Duffield et al. (2006)
	Mouse/retinopathy	Protects against neovascularization	Connor et al. (2007)
	Mouse/inflammatory pain	Inhibits spontaneous pain, heat, and mechanical hypersensitivity; selectively blocks TRPV1 and TRPA1-mediated pain	Xu et al. (2010) and Park et al. (2011)
	Mouse/obesity	Reduces inflammatory cytokines in adipose tissue macrophages; stimulates M2 macrophage differentiation; promotes resolution of adipose tissue inflammation	Titos et al. (2011)
	Mouse/T2 diabetes	Reduces macrophage accumulation in adipose tissue; ameliorates insulin sensitivity	Hellmann et al. (2011)
	Rats/post-operative pain	Reduces post-operative pain, tactile allodynia, and hyperalgesia	Huang et al. (2011)
	Mouse/pain	Attenuates agonist-induced and inflammatory pain behaviors; inhibits TRPA1, TRPV3, and TRPV4 receptors; does not affect basal sensitivity	Bang et al. (2010) and Xu et al. (2010)
	Mouse/acute lung injury	Blocks leukocyte infiltration and reduces cytokine levels in BALF	Wang et al. (2011)
	Mouse/corneal inflammation	Reduces leukocyte infiltration and hemangiogenesis	Jin et al. (2009)
AT-RvD1	Mouse/colitis	Reduces disease activity index, PMN number, and pro-inflammatory levels	Bento et al. (2011)
		Attenuates pain signals and behaviors by blocking TRPV3	Bang et al. (2012)
	Rats/arthritic pain	Possesses anti-hyperalgesic effects upon systemic administration. Decreases TNF-α and IL-1β production	Lima-Garcia et al. (2011)
Resolvin D2	Mouse/peritonitis	Blocks further PMN infiltration into the peritoneum	Spite et al. (2009a)
	Mouse/sepsis	Prevents hypothermia, decreases bacterial load in the blood and peritoneum, promotes survival	Spite et al. (2009a)
	Mouse/colitis	Improves disease activity index, weight loss, and colonic PMN infiltration. Reduces pro-inflammatory levels	Bento et al. (2011)
(Neuro)Protectin D1	Mouse/peritonitis	Inhibits neutrophil recruitment and regulates chemokine/cytokine production	Bannenberg et al. (2005) and Serhan et al. (2006)
		Promotes lymphatic removal of phagocytes; regulates T-cell migration; enhances CCR5 expression on apoptotic leukocytes	Ariel et al. (2005, 2006) and Schwab et al. (2007)
	Mouse/asthma	Protects from lung damage, airway inflammation, and hyper-responsiveness	Levy et al. (2007)
	Human/asthma	PD1 is generated in human asthmatic patients	Levy et al. (2007)
	Mouse/kidney ischemia/reperfusion	Protects from ischemia/reperfusion-induced kidney damage and loss of function; regulates macrophages	Duffield et al. (2006)
	Mouse/retinopathy	Protects against neovascularization	Connor et al. (2007)
	Rat/ischemic stroke	Inhibits leukocyte infiltration, NF-κB, and COX-2 induction	Marcheselli et al. (2003)
	Human/Alzheimer’s disease	Diminished PD1 production in human Alzheimer’s disease	Lukiw and Bazan (2008)
	Mouse/liver injury	Protects from necroinflammatory liver injury	Gonzalez-Periz et al. (2009)
	Mouse/Alzheimer’s disease	Downregulates inflammatory genes; reduces amyloidogenic Aβ42 cleavage; protects from apoptosis	Zhao et al. (2011)
Maresin-1	Mouse/peritonitis	Blocks PMN infiltration into the peritoneum	Serhan et al. (2009)
	Planaria/tissue regeneration	Stimulates tissue regeneration post surgical damage	Serhan et al. (2012)
	Mouse/pain	Reduces pain	Serhan et al. (2012)

Acute inflammation following tissue injury, surgery, or infections causes pain (Majno and Joris, 1996). Peripheral sensitization of primary sensory neurons is induced by inflammatory mediators released after tissue insults, such as bradykinin, prostaglandins, nerve growth factors (NGF), pro-inflammatory cytokines such as TNF-α, interleukin (IL)-1β and IL-6, and pro-inflammatory chemokines (Stein et al., 2009). The contribution of PGE_2_ and I_2_ led to the use of NSAID (e.g., naproxen, ibuprofen) and selective COX-2 inhibitors as analgesic. Since SPM are potent regulators of acute inflammation and pro-inflammatory mediators (including PGs, TNF-α, and IL-1β), and since COX-2 inhibitors are resolution toxic (Schwab et al., 2007), it was of interest to investigate whether SPM could control inflammation associated and chronic pain. The initial report from Svensson et al. (2007) on the antinociceptive actions of LXA_4_ was followed by further studies demonstrating that Rvs, PD, and also MaR1 have potent analgesic activities when administered both locally and systematically (Xu et al., 2010; Huang et al., 2011; Lima-Garcia et al., 2011; Park et al., 2011; Serhan et al., 2012). Notably, the exquisite potent actions of SPM, which proved as effective as morphine and COX-2 inhibitor NS-398 at much lower doses (Xu et al., 2010), occur without altering basal sensitive perception, unlike other anesthetics used to control pain during surgery. Hence it appears possible to resolve pain signaling as well as inflammation.

Complete resolution requires regeneration of destroyed tissues without affecting their functionality as in the case of fibrosis or scarring. Pro-resolving MΦ play key functions in tissue remodeling under both homeostatic (e.g., post parturition) and pathological (e.g., removal of microbes from infected tissues) conditions (Honn et al., 1989; Majno and Joris, 1996; Gordon, 2007). In this regard, SPM are of considerable interest in view of their roles in regulating MΦ activities. For instance, LX, Rv, and PD stimulate the non-phlogistic efferocytosis by MΦ (Godson et al., 2000; Schwab et al., 2007; Hong et al., 2008; Krishnamoorthy et al., 2010; Oh et al., 2011). In addition, RvD1 regulates MΦ accumulation in diabetic obese mice (Hellmann et al., 2011) and reduces arthritic pain (Xu et al., 2010; Lima-Garcia et al., 2011). Failure in the MΦ-driven pro-resolution program can support persistent inflammation associated with many human diseases, such as periodontitis. In keeping with this, recent reports indicate that MΦ from localized aggressive periodontitis have impaired phagocytosis and persistent inflammation that is rescued with RvE1 (Fredman et al., 2011). In addition to enhancing MΦ phagocytosis, MaR1 biosynthesized *in vivo* during tissue injury repair also accelerated tissue regeneration in planaria (*D. tigrina*) after surgical head removal (Serhan et al., 2012). Of note, these actions of MaR1 were inhibited by PTX, indicating the involvement of GPCR and related signaling in this process (Serhan et al., 2012).

## miRNAs in Resolution Circuits

**Figure 6:**
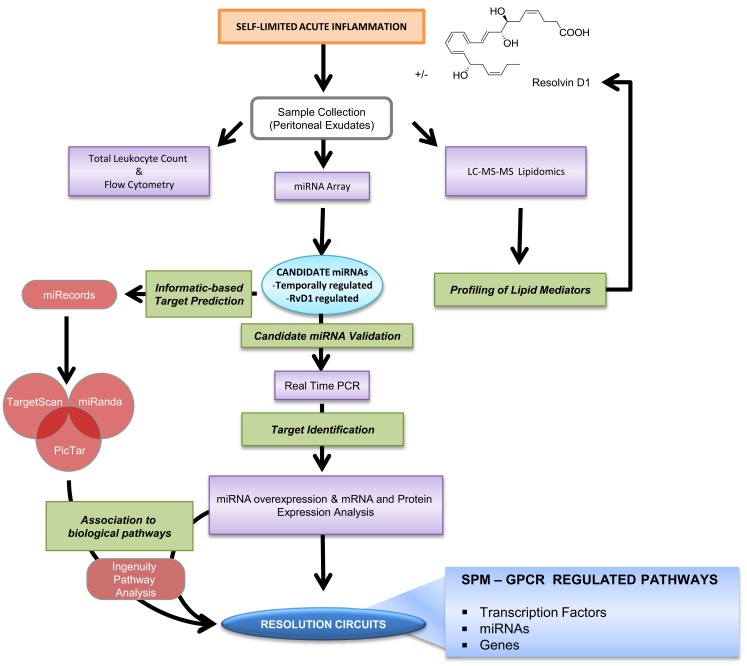
**Strategy for identification of RvD1-GPCR circuits**. Self-limited zymosan-stimulated mouse peritonitis was used to obtain resolving exudates that were collected 4, 12, 24, and 48 h after zymosan A injection for temporal and differential analysis of leukocyte counts and phenotype, miRNA real-time PCR analysis, and lipidomics as in Recchiuti et al. (2011) and Krishnamoorthy et al. (2012). Candidate miRNAs, defined as those that were temporally and/or RvD1-regulated, were confirmed using bioinformatics and prediction of target genes, real-time PCR, as well as overexpression in human MΦs. The Ingenuity Pathway Analysis Software database was used to identify circuits of molecules and biological functions controlled by RvD1-regulated miRNAs and target genes (see text; Recchiuti et al., 2011 for analysis details).

**Figure 7:**
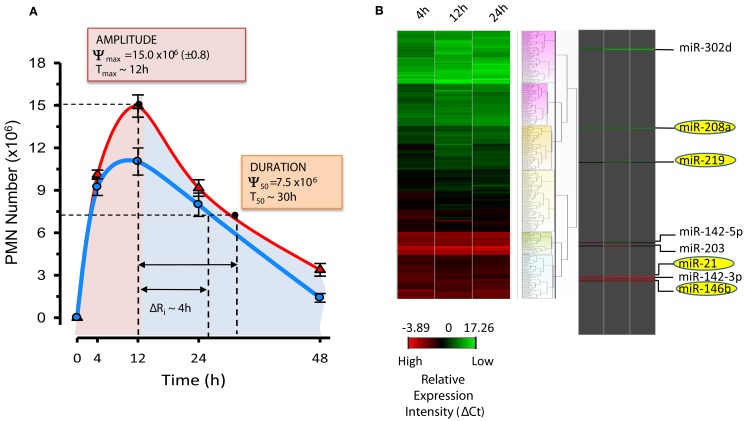
**Resolution indices and miRNA signature of resolution**. **(A)** Resolution indices were calculated as in (Bannenberg et al., 2005) using PMN numbers in peritoneal exudates from mice treated with zymosan alone (red line) and zymosan plus RvD1 (blue line) at indicated time intervals: *T*_max_, time of maximum PMN infiltration (Ψ_max_); *T*_50_, time to achieve 50% reduction in PMN number (Ψ_50_) from Ψ_max_; *R*_i_, resolution interval (*T*_50_ − *T*_max_; time interval between *T*_max_ and *T*_50_). RvD1, administered at the beginning of peritonitis, significantly lowers amplitude (i.e., Ψ_max_) and duration (*T*_50_) of PMN infiltration, shortening the *R*_i_ by ∼4h. **(B)** Heat map cluster represents relative expression of ∼300 mouse miRNAs from resolving exudates determined with real-time PCR after normalization with housekeeping small RNAs (snoRNA251, snoRNA202, snoRNA142, and Rnu6). Relative expression intensities are indicated in a green-red color code based on ΔCt expression values (*n* = 3 mice/group). Temporal and RvD1-regulated miRNAs are highlighted.

Results from the Serhan laboratory at Brigham and Women’s Hospital-Harvard Medical School demonstrated for the first time that SPM are operative in resolution and act locally to control leukocyte trafficking, regulate chemical mediators (e.g., cytokines, chemokines, and lipid autacoids), and expedite the return to homeostasis. Since microRNA (miRNAs) have emerged as the fine tuners of many cellular processes, including immune responses and cancer, it was of interest to investigate whether they also played roles in resolution and SPM-regulated specific miRNA as part of their mechanisms of action. To identify a miRNA signature of resolution in self-resolving exudates, a strategy with resolving exudates from murine peritonitis was used (Figure 6). Zymosan A particles from *S. cerevisiae*, a Toll-like receptor 2 and 4 ligand, were injected i.p. and peritoneal exudates collected to monitor temporal changes in both leukocyte numbers and composition. Rapidly after zymosan injection, leukocytes infiltrated the peritoneal cavity during the onset phase of acute inflammation (4 h), reaching a maximum (∼22.0 × 10^6^) at ∼12 h and declined at 24–48 h. Differential analysis with flow cytometry confirmed that the majority of leukocytes in exudates at 4 and 12 h were PMN (Ly-6Ghigh/CD11b^+^; ∼10.0 × 10^6^ cells at 4 h and ∼15 × 10^6^ cells at 12 h). PMN number declined during the resolution phase 24 and 48 h after initiation of peritonitis. Conversely, resident peritoneal MΦs (F4/80^+^cells), which represent the main leukocyte population in naive mice peritonea (Winyard and Willoughby, 2003), were not detected in exudates at 4 h. Monocytes (Ly-6G^low/^CD11b^high^ cells) gradually increased at 12 h and differentiated into MΦs, consistent with their pro-resolution functions (Gordon, 2007), reaching ∼60% of exudate leukocytes at 48 h. Because it is important to define resolution in unbiased terms, resolution indices, introduced by Bannenberg et al. (2005), were calculated (i.e., Ψ_max_ ∼ 15.0 × 10^6^; *T*_max_ ∼ 12 h; Ψ_50_ ∼ 7.5 × 10^6^; *T*_50_ ∼ 30 h; *R*_i_ ∼ 18 h; Figure 7) to characterize the resolution phase and determine temporal changes in miRNA expression during this time interval. Hierarchical clustering grouped the ∼300 miRNAs examined into distinct clusters based on their relative abundance at the different time intervals, indicating that specific miRNAs are temporally regulated during acute inflammation and its natural self-limited resolution.

In these studies, miR-21, miR-146b, miR-208a, and miR-219 were significantly regulated at 12 and 24 h compared to 4 h (Figure 7), suggesting a role in resolution. For instance, miR-21 proved to be critical for the production of anti-inflammatory IL-10 in experimental peritonitis (Sheedy et al., 2010), corroborating our findings. Next, resolution indices were used to pinpoint RvD1 actions in resolution. RvD1, administered in its pro-drug carboxy methyl ester form, significantly lowered the Ψ_max_ (from 15.0 to 11.5 × 10^6^), and accelerated or shortened the *R*_i_ by ∼4 h. Quantitative real-time PCR analyses carried out with exudates from zymosan- and zymosan plus RvD1-treated mice revealed that RvD1 temporally controls the specific sets of pro-resolving miRNAs miR-21, miR-146b, miR-208a, and miR-219 (coined *resolution miRs*) in exudates *in vivo*. For translation to humans, actions of RvD1 on these miRNAs were assessed in ALX/FPR2 and DRV1/GPR32-overexpressing isolated human cells, namely MΦ, the master regulators of resolution. Notably, RvD1 also regulated miR-21, -146b, -208a, and 219 in a GPCR-dependent manner. In order to identify target molecules of RvD1-GPCR-regulated miRs, we overexpressed miR-146b, -208a, and -219 in human MΦ for real-time PCR analysis to assess which mRNAs were significantly regulated.

**Figure 8:**
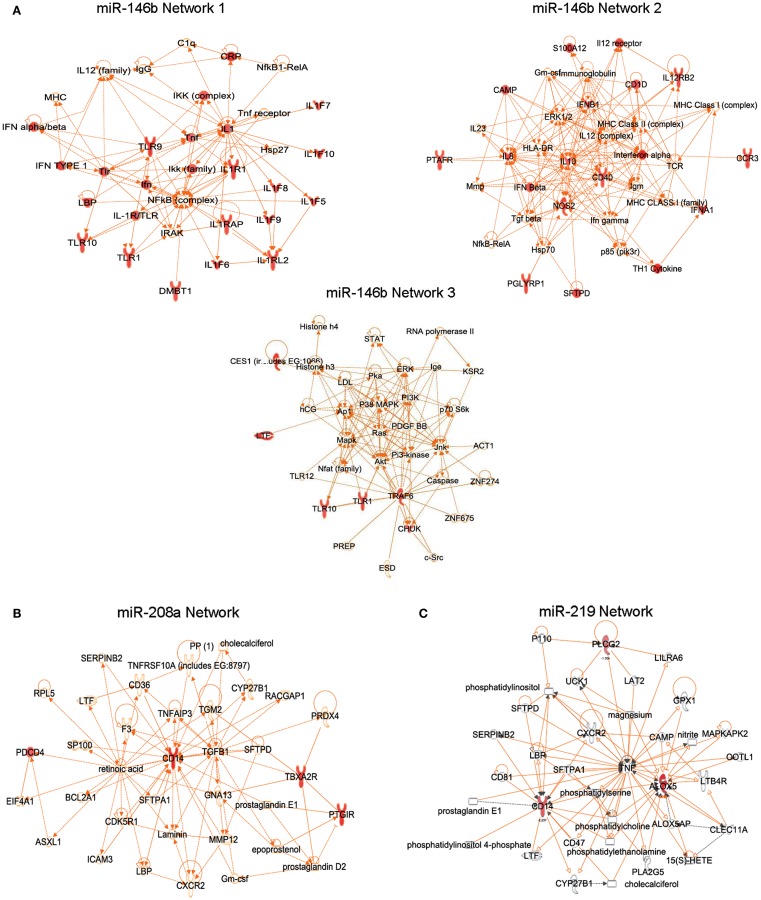
**Translation of RvD1-dependent miRNA circuits in human cells**. RvD1-GPCR gene networks connecting target genes of miR-146b **(A)**, miR-208a **(B)**, and miR-219 **(C)**. Genes that are significantly down regulated in each network are indicated in red.

Since miRNAs can regulate mRNAs and protein levels of hundreds of genes involved in biological processes (Bartel, 2009), miR-target genes were clustered using the Ingenuity Pathway Analysis knowledge database based on their physical and/or functional interactions, creating the first identified RvD1-GPCR-regulated gene networks involved in inflammation and resolution. For instance, miR-146 networks 1 and 3 included genes of the NF-κB activation pathway (e.g., IκB kinase and tumor necrosis factor receptor-associated factor 6) and innate response to pathogens (e.g., Toll-like receptors, S100 protein, C-reactive protein, peptidoglycan recognition protein; Figure 8A). Several cytokines and chemokines (IL-8, 10, 12, interferon-α and β) belonged to the miR-146b network 2 (Figure 8A). NF-κB is a critical transcription factor involved in regulation of cell functions in inflammation and resolution (Lawrence et al., 2001). Of interest, RvD1 in human monocytes reduces the nuclear translocation of NF-κB, TNF-α induced phosphorylation of IκB (Recchiuti et al., 2011), counteracts NF-κB activation in ALX/FPR2 and DRV1/GPR32 recombinant cells (Krishnamoorthy et al., 2010), dampens acute inflammation in murine dorsal air pouches evoked by local administration of TNF-α (Serhan et al., 2002), and downregulates IKK levels in murine peritonitis (Recchiuti et al., 2011). Therefore, regulation of miRNAs and the TNF-α-NF-κB axis seems to be a key component in the RvD1-GPCR downstream signaling network.

The miRNA miR-208a downregulates CD14, CD40 ligand, PGI2 receptor, thromboxane A2 receptor, and programmed cell death (Recchiuti et al., 2011; Figure 8B), a tumor suppressor molecule that acts as a translational repressor of IL-10 (Sheedy et al., 2010), consistent with the existence of a seed region for miR-208a in the 3′ UTR of PDCD4. Notably, in self-limited peritonitis, RvD1 reduced PDCD4 and increased IL-10 production, providing *in vivo* correlates of RvD1-miR-dependent gene regulations.

The miR-219 network includes CD14 and 5-LO (Figure 8C), a key enzyme for the biosynthesis of LTs, and SPMs. In addition, a significant reduction in 5-LO protein levels and LTB_4_ production was found in human MΦ overexpressing the RvD1-regulated miR-219, translating findings in mouse peritonitis to the human MΦ cell system (Recchiuti et al., 2011). Endogenous regulatory mechanisms of 5-LO are of wide interest given the important roles of LTs, LXs, and Rvs in inflammation and resolution (Samuelsson, 1983; Serhan et al., 2002). In addition to transcriptional regulation by cytokines and growth factors (Radmark et al., 2007), miR-219 can provide a rapid mean to balance the abundance of 5-LO protein in cells under dynamic conditions such as during inflammation.

**Figure 9:**
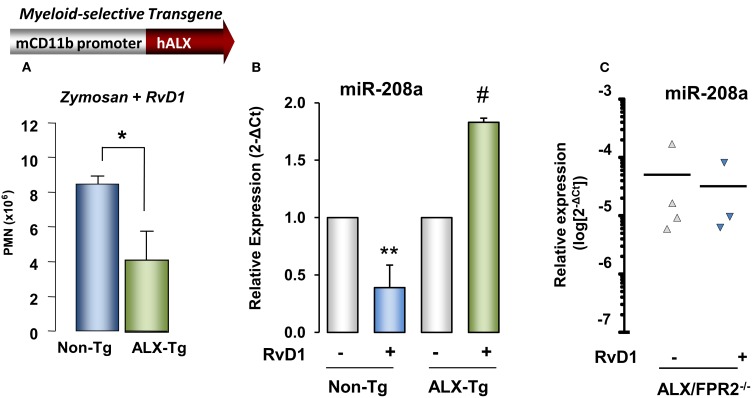
**Evidence for ALX/FPR2-RvD1-dependent miRNA pathways *in vivo***. Reduction in **(A)** PMN infiltration by RvD1 is significantly higher in myeloid-driven human ALX-transgenic (Tg) mice challenged with zymosan (1 mg/mouse, i.p., 24 h) than non-Tg littermates. Results are mean ± SEM from three mice/group (*P < 0.05 vs. non-Tg). **(B)** Relative expression of miR-208a determined in resolving exudates 24 h after zymosan alone or zymosan plus RvD1 administration in hALX-Tg or non-Tg littermates (mean ± SEM, *n* = 3 mice/group; **P < 0.05 vs. non-Tg zymosan group; #, *P* < 0.05 vs. hALX-Tg zymosan group). **(C)** miR-208a expression in peritoneal exudates (24 h) from ALX/FPR2 knockout mice treated with zymosan alone or zymosan plus RvD1.

RvD1 actions were also tested in genetically engineered mice in order to obtain further evidence of ALX/FPR2-dependent action. Transgenic mouse colonies in which human ALX/FPR2 gene was placed under control of a CD11b promoter that drives transgene expression in mature murine myeloid cells were created (Devchand et al., 2003). Interestingly, total peritoneal leukocytes at 24 h peritonitis were significantly lower in ALX/FPR2 transgenic mice treated with zymosan alone compared to non-transgenic littermates, and RvD1 administration resulted in a further significant decrease (∼53% reduction) in peritoneal exudate leukocyte numbers in ALX/FPR2 transgenic mice compared to non-transgenic littermates (Figure 9). Quantitative PCR analysis of miRNAs isolated from peritoneal lavages collected 24 h post injection of zymosan showed that RvD1 significantly upregulated identified resolution miRs miR-208a and miR-219 *in vivo* (Recchiuti et al., 2011), whereas it does not appear to regulate miR-21, miR-146b, and miR-302d (Figure 9). To further investigate ALX/FPR2-dependent actions of RvD1 *in vivo*, it was next tested whether RvD1 can regulate some of these miRNAs in ALX/FPR2 knockout mice, where LXA_4_ did not regulate leukocyte trafficking (Dufton et al., 2010). In ALX/FPR2^−/−^ mice, RvD1 did not regulate PMN infiltration, consistent with recent results (Norling et al., 2012), nor did it significantly alter miR-208a or miR-219 expression at 24 h (Figure 9).

Taken together, these results indicate that RvD1 controls leukocyte infiltration and specific resolution phase miRs in acute inflammation in an ALX/FPR2-dependent manner in mice and DRV1/GPR32-dependent in isolated human cells.

## Summation and Conclusion

It is now eminently clear that the acute inflammatory response of the host is a highly coordinated defensive response and its complete resolution is the ideal outcome. This ideal outcome is achieved in self-limited inflammation by activation of endogenous resolution programs that are governed in part by resolution phase local chemical mediators. Excessive inflammation can lead to chronic disorders and host damage. LM from AA, such as PGs and LTs, can amplify the inflammatory response, and PGE_2_ and D_2_ initiate the “class switch” that leads to the biosynthesis of LXA_4_, the first member of the new genus of SPM. SPM include several families of LM such as E-and D-series Rv (neuro)PD, and the youngest family, the MaR, which are biosynthesized from essential ω-3 fatty acids. Of interest in experimental animal models of disease, each of the SPM can accelerate the resolution process when administered *in vivo*, and their bioactions are highly stereospecific, GPCR-mediated, and exerted at low doses. SPM also reduce pain and promote tissue regeneration, the ultimate goal of complete return to homeostasis. Results from the first human clinical Phase I and II trial with a Rv analog appear encouraging and can open new opportunities for resolution pharmacology based on endogenous mediators to terminate inflammation and treat inflammation-related diseases. We trust that more human trials will be launched to test the notion that stimulating resolution mechanisms can improve disease and health status.

## Conflict of Interest Statement

Charles N. Serhan is an inventor on patents [resolvins] assigned to BWH and licensed to Resolvyx Pharmaceuticals. Charles N. Serhan is a scientific founder of Resolvyx Pharmaceuticals and owns equity in the company. Charles N. Serhan’s interests were reviewed and are managed by the Brigham and Women’s Hospital and Partners HealthCare in accordance with their conflict of interest policies. Antonio Recchiuti declares no conflict of interest.
